# Direct Laser Irradiation and Modification of 2D Te for Development of Volatile Memristor

**DOI:** 10.3390/ma16020738

**Published:** 2023-01-12

**Authors:** Genwang Wang, Yanchao Guan, Yang Wang, Ye Ding, Lijun Yang

**Affiliations:** 1 Key Laboratory of Microsystems and Microstructures Manufacturing, Ministry of Education, Harbin Institute of Technology, Harbin 150001, China; 2 School of Mechatronics Engineering, Harbin Institute of Technology, Harbin 150001, China

**Keywords:** laser irradiation, tellurene, defects, oxides, volatile memristor

## Abstract

Laser irradiation, as a kind of post-fabrication method for two-dimensional (2D) materials, is a promising way to tune the properties of materials and the performance of corresponding nano-devices. As the memristor has been regarded as an excellent candidate for in-memory devices in next-generation computing system, the application of laser irradiation in developing excellent memristor based on 2D materials should be explored deeply. Here, tellurene (Te) flakes are exposed to a 532 nm laser in the air atmosphere to investigate the evolutions of the surface morphology and atom structures under different irradiation parameters. Laser is capable of thinning the flakes, inducing amorphous structures, oxides and defects, and forming nanostructures by controlling the irradiation power and time. Furthermore, the laser-induced oxides and defects promote the migration of metal ions in Te, resulting in the formation of the conductive filaments, which provides the switching behavers of volatile memristor, opening a route to the development of next-generation nano-devices.

## 1. Introduction

It is notable that 2D materials, such as graphene, black phosphorene(BP), transition metal dichalcogenides (TMDs), and hexagonal boron nitride (h-BN), have aroused intensive attention as promising candidates for next-generation transistors, photodetectors, sensors, memristors, etc. [1,2,3,4]. With achievements of synthesizing large-scale and high-quality 2D materials by mechanical exfoliation, chemical vapor deposition (CVD), physical vapor deposition (PVD), and molecular beam epitaxy (MBE) [5], wafer-scale functional devices and circuits were fabricated and exhibited promising properties [6,7]. These demonstrate that 2D materials have potential for high-performance devices and solving the bottleneck problems of traditional silicon-based devices in the future. In particular, memristors based on 2D materials also have promising and gorgeous performances, including lager switching ratios (SR), low energy consumption, and excellent stability for in-memory and neuromorphic computing, which are promising solutions for next-generation computing systems [8,9,10]. Besides the exploration of advanced synthesis methods, post-fabrication of 2D materials are also critical ways to further tune and improve properties of materials and corresponding nano-devices. For instance, electron beam can write doping patterns with high-resolution in graphene and MoS_2_, so that devices exhibit lower subthreshold swing, higher carrier concentration, and higher mobility, compared to the pristine [11]. Electron beam irradiation also induces local phase transition, which leads to resistive switching in memristors based on PdSe_2_ [12]. Etching techniques based on plasma, reactive gas, and thermal annealing are capable with thin, oxidized, patterned 2D materials but can also form defects and nanostructure in 2D materials [13,14]. Laser irradiation is also considered as a flexible and clean approach to realize layer control, phase transformation, pattern, oxidation, and connection in nanomaterials [15,16,17]. After laser exposure, materials and devices present some new advanced properties, such as improvement of conductivity and hole mobility of PdSe_2_ [18], higher drain current modulation of laser-irradiated BP than pristine [19]. Therefore, laser modification of 2D materials provides a fertile library for improving the performance of advanced nano-devices. In addition, laser is also capable of modifying 2D materials and ultrathin films to induce or promote switching performance of corresponding devices, indicating the enormous potential of the laser irradiation in the application of fabricating memristors [20,21,22].

Additionally, 2D Te, as a semiconductor material is currently under the spotlight due to unique properties, including tunable bandgap, large strain limit, and high mobility. It can be a promising candidate for development of excellent transistors, photodetectors, sensors, and flexible devices [23,24,25,26]. Moreover, the Van der Waals heterostructures based on Te also demonstrate high rectification ratio and photo/dark current ratio, which offers an opportunity to fabricated diodes and optoelectronic devices with promising performances [27]. Previous investigations have revealed that laser-induced preparation of nanocrystals in Te flakes, resulting in improvement of optical properties [28]. Hence, in order to have deep insight into the effects of laser on Te flakes and further give a guideline for tuning electrical properties of materials and nano-devices, more investigations about the evolution of morphology, atom structures, and elements contents under different laser parameters are indispensable. In addition, as other materials have demonstrated the switching behaviors of memristors, the development of memristor based on 2D Te is necessary and of agency. Thus, the exploration of the control of properties of 2D Te flakes which are processed by other methods (especially the laser irradiation with selective area) can provide new promising ways to develop memristor with outstanding performance.

Here, Te flakes are exposed to 532 nm laser with different power and irradiation time in air conditioning to investigate the evolution of the surface morphology, atom structure, and the element composition. The surface morphology and the Raman spectra of laser-irradiated flakes demonstrate that high laser power is capable of ablating the materials to get thinner flakes. However, lower laser power results in protrusions in nano-scale on the surface. The formation of such nano-protrusions can be attributed to laser-induced oxides, amorphous materials, and nano-structures on the surface. Moreover, the size of nano-protrusions can be adjusting by varying irradiation time. The high-resolution transmission electron microscope (HR-TEM) and energy dispersion X-ray spectrometry (EDS) have confirmed the evolution of “ablation, formation of amorphous oxide structures, and generation of defects and oxides” in the Te flakes as the power decays. Hence, using suitable laser parameters, defects, and oxides are induced in Te without destroying the surface to develop two terminal vertical memristors. The laser-irradiated Te flakes exhibit properties of digital-type volatile memristor, while pristine flakes have no switching performance. Thus, the laser-induced defects and oxides promote the migration of Ti metal ions resulting the formation of conductive filaments, which leads to the switching between high resistance state (HRS) and low resistance state (LRS). Hence, memristor based on laser-irradiated Te provides opportunity to in-memory computing.

## 2. Materials and Methods

### 2.1. Synthesis of Tellurium Nano-Flakes

Tellurium nanoflakes were synthesized by a solution-grown method based on previous reports [23,25] with slight modifications. In a typical procedure, 230 mg Poly(vinylpyrrolidone) (PVP, average MW 58000) and 46 mg sodium tellurite (Na_2_TeO_3_) were dissolved in 16 mL deionized (DI) water in turn. The mixed solution was stirred for 30 min at room temperature for complete dissolution. Then, hydrazine monohydrate (N_2_H_4_·H_2_O, 80%) and ammonium hydroxide solution (NH_3_·H_2_O, 28%) were placed into above solution, which was poured into a 25 mL Teflon-lined stainless-steel autoclave. After well sealing and enough shaking to form a homogeneous solution, the autoclave was putted in an oven and heated to 180 °C from room temperature and maintained at 180 °C for 15–20 h. Then, the autoclave was cooled to room temperature naturally. The products were transformed into plastic tubes and washed at least 3 times with DI water by centrifugation at 7000 r.p.m. for 20 min. The final solution has a silver-gray color and are mixed with DI water. Then, the solution was spin-coated on a substrate for further experiments. As the synthetic solution also contains nanowires and nanoflakes, the sample was ultra-sonicated in acetone for 5 min to remove some nanowires. Finally, the substrate with tellurium nanoflakes was washed with DI water and dried by nitrogen gas for next experiments.

### 2.2. Devices Fabrication and Characterization

The bottom electrodes were firstly fabricated by electron-beam lithography (EBL) and the e-beam evaporation [6,8]. The p-doped Si substrate with 285 nm SiO_2_ was spin-coated by poly(methyl methacrylate) (PMMA) A4 950 photoresist. Then, the photoresist was sequentially baked, exposed by electron-beam and developed by mixed methyl isobutyl ketone (MIBK) and isopropanol(IPA) solution with ratio MIBK: IPA = 1: 3. Next, the metal Ni/Au (5 nm/25 nm) were evaporated in turns via e-beam evaporation. After that, acetone and DI water were used to perform the lift-off process to remove the PMMA layer and the deposited metal on it. After the fabrication of bottom electrodes, the Te flakes were spin-coated or transferred on electrodes using Polydimethylsiloxane (PDMS) films and the 2D materials transfer stage. Then, the top electrodes Ti/Au (20 nm/60 nm) were fabricated by EBL and e-beam evaporation. The electrical measurements were carried in ambient conditions at room temperature using a semiconductor parameter analyzer (B1500A, Keysight, Santa Rosa, CA, USA) and a probe station with tungsten tips.

### 2.3. Simulations of Exfoliation of Te Flakes and Formation of Native Defects

Simulations were performed based on first-principles density functional theory (DFT). For Te flakes, the generalized gradient approximation of the Perdew–Burke–Ernzerhof (PBE) exchange correlation function, projector augmented-wave (PAW), pseudopotential, and cut-off energy of 500 eV were adopted. To build the atom structure of layered Te, the vacuum space of 20 Å was introduced along the direction of thickness. A k-mesh of 15 × 15 × 1 sampling was used to relax all structures until the energy in electronic self-consistent field (SCF) iterations and the ionic Hellmann–Feynman forces were lower than 1 × 10^−6^ eV/atom and 0.01 eV/Å, respectively. The exfoliation energies *E*_exf_ were calculated by

(1) $$E\mathrm{exf}(n)=\frac{E\mathrm{layer}(n)-Eflake\;n/m}{S}$$

where *E*_layer_(*n*) is the energy of the unit cell of exfoliated *n*-layer Te, *E*_layer_ is the energy of the bulk Te with *m* layers, *S* is the in-plane area of the bulk unit cell [29]. The formation energy of defects in Te is defined as

(2) Ef=Edefects−Epristine±μTe

where *E*_defects_(*n*) and *E*_pristine_(*n*) are the energy of defective and pristine monolayer Te with a 3 × 3 × 1 supercell, *μ*_Te_ is the chemical potential of a Te atom obtained from bulk tellurium, and ± denotes vacancy (+) or interstitial (−).

## 3. Results and Discussion

### 3.1. Preparation and Characterization of Te Nano-Flakes

**Figure 1:**
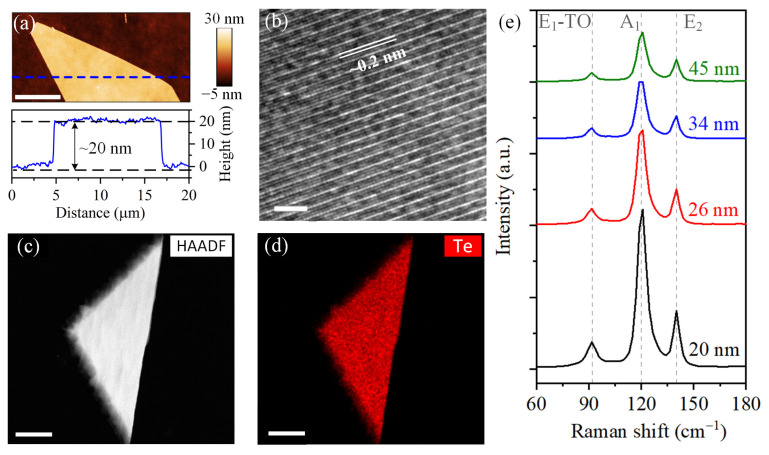
Synthesized Te flakes and material characterization. (**a**) AFM image (the top inset image) of a Te nano-flake with thickness of ~20 nm and the corresponding height profile (the bottom inset image, the position is shown by the blue dotted line in the top inset image), scale bar is 5 μm; (**b**) HR-TEM image of atom strictures, scale bar is 4 nm; HAADF image (**c**) and EDS mapping image (**d**) of Te element; (**e**) Raman spectra of Te flakes with different thicknesses.

**Figure 2:**
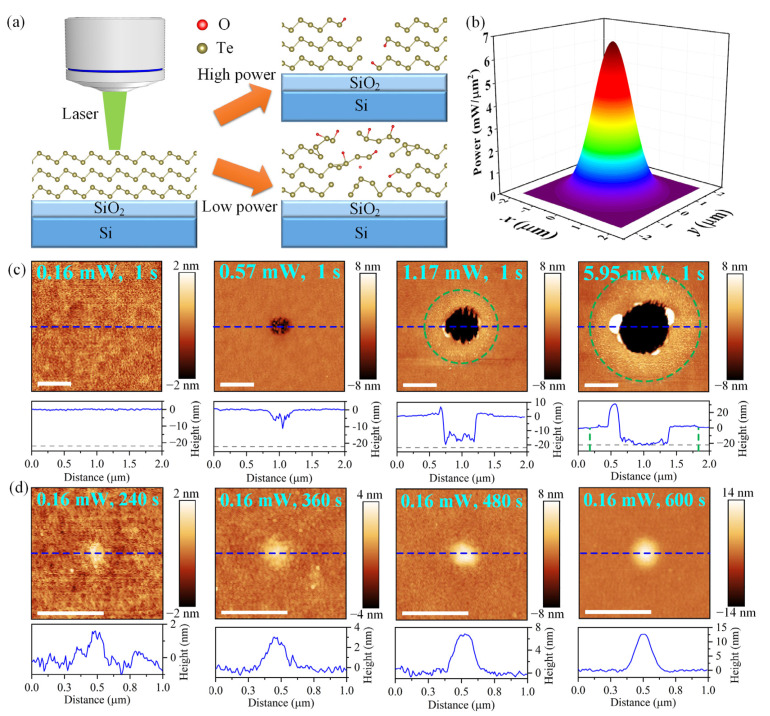
Modification of the Te flake irradiated by laser with different power and time. (**a**) Schematic of the laser irradiation process; (**b**) schematic of the laser beam with Gaussian distribution of power density; AFM images (top inset images) of Te nano-flakes irradiated by laser with different power (**c**) and time (**d**) and the corresponding height profiles (bottom inset images, the positions are shown by the blue dash lines in the top inset images, the gray dotted lines indicate the thickness of the flake, the green dotted circles indicate the laser irradiation area), scale bar is 500 nm.

In a typical experiment, Te nano-flakes are synthesized by a hydrothermal method then spin-coated or transferred on a SiO_2_/Si substrate (Details are shown in Materials and Methods). The synthesized Te flakes have thickness ranging from 20 nm to 100 nm and lateral lengths from 10 to 100 μm, which can be acquired by adjusting the reaction time and the Na_2_TeO_3_/PVP ratio. A Te flake with thickness of ~20 nm is shown in Figure 1a. The elements and atom structures of solution-synthesized Te flakes are confirmed by HR-TEM and EDS. Figure 2b presents the measured lattice constants are ~0.2 nm and ~0.6 nm, which are assignable to the {$1\bar{2}10$} and {0001} planes, respectively, and consistent with previous experimental and calculation investigations [25,26]. The high-angle annular dark field (HAADF) image and EDS mapping image in Figure 1c and 1d have confirmed that the nano-flake consists of Te atoms. Hence, such atom structure and element components have proved that the synthesized nano-flakes are tellurene. Figure 1e demonstrates that the Te flakes which has thickness larger than 20 nm have three main peaks located around at 92 cm^−1^, 120 cm^−1^ and 140 cm^−1^, corresponding to the vibration modes of E_1_-TO, A_1_, and E_2_, respectively. Figure S1 presents the variations of the fitting locations and intensities of Raman peaks. The locations have no obvious shift regardless of the thickness. However, as the thickness decrease, the peaks have an increase in the intensities. Thus, the intensities of the three characteristic peaks can be used to approximately estimate the thickness of Te flakes, and both AFM images and Raman spectra confirm the successful preparation of Te nano-flakes.

### 3.2. Laser-Induced Ablation, Oxidization, and Defects in Te Nano-Flakes 

Te nano-flakes with thicknesses ranging from 20 nm to 23 nm were selected to investigate the effects of laser irradiation using a Raman spectrometer with a 532 nm laser source. The schematic of laser modification process is shown in Figure 2a. The nano-flakes were irradiated by laser in the air atmosphere and at room temperature. The power and spot size of the laser can be adjusted by choosing different power filters and objective lenses, respectively. As will be discussed in detail below, the Te flake is extremely sensitive to laser power and irradiation time. The high power leads to the ablation and thinning of the flake, while low laser power can induce defects and oxides in flake and formation of nano-structures on the surface as the irradiation time increases. Here, the 100× objective lens was chosen to get a laser spot with diameter about 1.5 μm. The distribution of the laser power density can be formulated by the Gaussian function, which has degenerative power density from the center of the spot to the edge, as the schematic shows in Figure 2b. To reveal the effects of laser power on the surface morphology and atom structures, the flakes were exposed for *T* = 1 s to laser with different power *P* by choosing different power filters. After the irradiation, Raman spectra were obtained using a safety laser power (~17 μW) to avoid further degradation of materials. The surface morphology (see Figure 2c) were measured by AFM in a tapping mode. The power of 0.16 mW has negligible influence on the surface of Te, indicating lower power has no effect on the materials. The laser results in the ablation of materials and the formation of nano-structures as the power increases to 0.57 mW. Furthermore, higher laser power causes a deeper ablation hole with lager diameter in Te flakes. Besides, in the laser irradiation area outside the ablation area (the green dotted circle), the Te flake has become thicker and demonstrated a rougher surface. Such a phenomenon can be attributed to the effect of low power density area of the laser beam. The power of 5.95 mW nearly removes all the Te materials in the ablation area. Therefore, a power higher than 5.95 mW will cause more ablation. Moreover, recasts of melted materials are observed at the edge of the ablation hole. Thus, high laser power causes the ablation of Te flake.

To have deeper insight into the modification induced by low power, Te flakes were exposed to lasers with powers of 0.16 mW and prolonged irradiation times. Figure 2d exhibits the surface morphology of Te flakes after the laser irradiation with different exposure time of 240 s, 360 s, 480 s, and 600 s. It is apparent that a laser-induced protrusion with diameter of ~200 nm is observed at the center of the laser spot. The height of the protrusion can be adjusted by varying the irradiation time. For example, after exposing a Te flake to low-power laser for 600 s, a protrusion with height of ~12 nm is acquired. Previous research has demonstrated laser-irradiated 2D PdSe_2_ and MoS_2_ also had nano-structures on the surface (the heights of the nano-structures are about 2–4 nm). The nano-structures were attributed to the ablation of nanoparticles from the flake and the re-deposition of these on the surface [18,30,31]. Compared to the nanoparticles on the surface of PdSe_2_ and MoS_2_, the laser-irradiated Te flake has a high protrusion, which is not similar to re-deposited nanoparticles. The generation of the protrusions could be attributed not only to the ablation and adsorption of Te nanoparticles, but also the formation of nano-structures on the surface, for example nanocrystals [28] and the deformation of materials. Moreover, the size of the laser-induced protrusion can be adjusted by varying the size of laser spot using objective lens with low magnification. For instance, the lateral size of the protrusion can be increased to 500 nm using a 50× objective lens, as Figure S2 shows. Hence, the laser with low power can lead to the destruction of the pristine Te flake and the formation of nano-structures on the surface.

**Figure 3:**
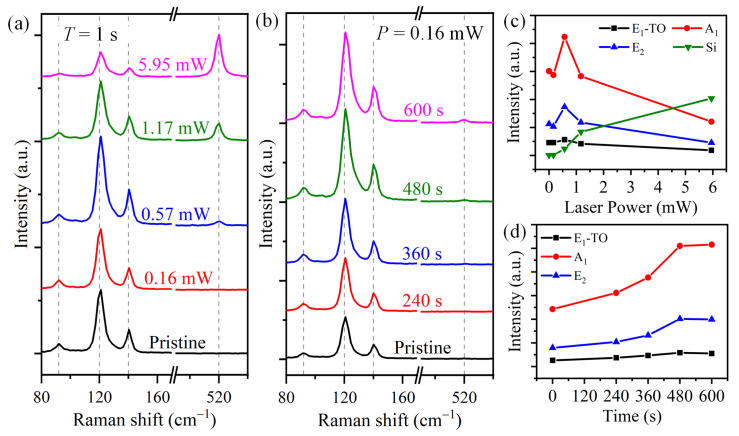
Changes of Raman spectra in Te flakes irradiated by laser. Raman spectra of Te flakes under different power (**a**) and irradiation time (**b**) and corresponding fitting intensities of peaks (**c**,**d**), respectively.

The evolution of the Raman spectra of Te flakes irradiated by laser with varying power and time is investigated to confirm the effect of laser on atom structure. As Figure 3a shows, under the different laser power, Te nano-flakes demonstrate negligible changes in the positions of the three characteristic peaks E_1_-TO, A_1_ and E_2_ peaks (shown in Figure 1e) and have no new peak. In contrast, the laser-irradiated Te flake has a variation of peak intensity depended on power, as the fitting intensities are showed in Figure 3c. The power of 0.16 mW has no influence on the Raman spectrum, indicating no effect on the Te flake, which is consistent with the AFM results. Therefore, it can be concluded that a lower power than 0.16 mW has no influence on Te flake. However, as the power increases, the three characteristic peaks increase, followed by a drastic decrease in the intensities, while the intensity of the Si peak is increased. Such variation means that local modification and degradation of Te materials are induced by laser sequentially. Higher laser power leads to further ablation, which consistent with the results in Figure 2c. Figure 3b,d present he Raman spectra of the Te flakes which were measured after the laser irradiation with power of 0.16 mW and different time to further reveal the influence of low-power lasers on materials. Similarly, Te flakes demonstrate the invariable positions of the three characteristic peaks and the stronger intensities, as the irradiation time prolongs. Besides, it should be noted that only the flake irradiated by laser for 600 s has a very weak Si peak. The weak Si peak could be attributed to the destruction of pristine Te atom structure. As the irradiation time extends, laser induces more destruction of surface atoms, formation of thicker protrusions, and more defects in deeper materials. Therefore, the irradiated materials cannot have dense atom structure like pristine Te, resulting in the observation of the weak Raman peak from substrate. Pervious research also demonstrated the stronger Si peak after the formation of laser-induced nanostructure on the surface of Te flake [28]. Hence, the Raman spectra have confirmed again that the laser irradiation with low power and long exposure can result in the modification of atom structures without thinning of the Te flake.

**Figure 4:**
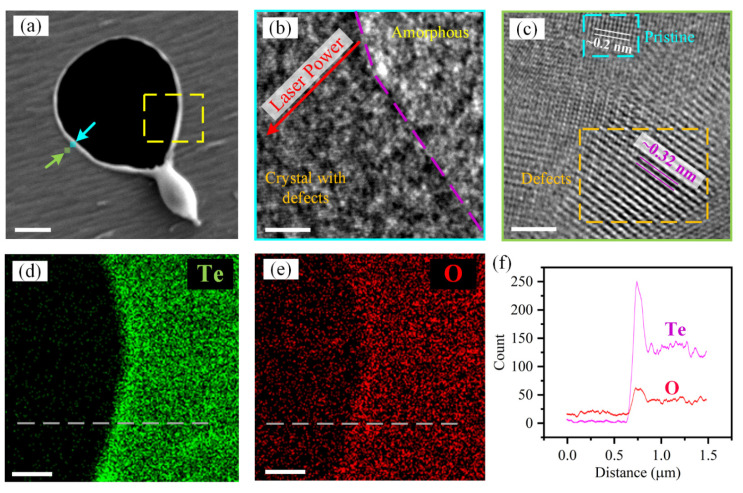
STEM and EDS measurement results of a laser-irradiated Te flake. (**a**) low-magnification image of laser-irradiated Te, scale bar is 1 μm; variations of atom structure of Te depended on laser power, the measurement locations are shown by the light blue (**b**) and orange (**c**) squares, respectively in the (**a**), scale bar is 2 nm; ESD mapping images of Te (**d**) and O (**e**) elements at the location shown by the gold dash rectangle in the (**a**), scale bar is 500 nm. (**f**) the line-scan EDS at the location shown by the gray dash lines in (**d**,**e**).

A deeper insight into the effect of laser irradiation on the atom structure of Te flake was acquired by using scanning transmission electron microscopy (STEM) and EDS. Figure 4a presents the low-magnification STEM image of laser-irradiated Te flake. Since the laser power decays from the center of the laser spot to the outside, the Te flake is ablated and slightly modified in turns. Moreover, the recast of melted materials is also observed, consisting with the AFM images shown in Figure 2c. To reveal the variation of atom structures depended on lase power, the magnified HR-TEM images of the modified Te (the locations are annotated in the blue and orange squares and arrows in Figure 4a) are exhibited in Figure 4b,c. Under the relative higher power, the Te crystal is ablated, and the materials are transformed into amorphous structures. As the power descents (the red arrow presents the direction), despite no crystal structure of pristine Te is showed, obvious Te atoms with abundant defects are detected. Such atom structure indicates that the moderate power is capable of inducing abundant defects instead of amorphous materials. Furthermore, as Figure 4c shows, after exposed by lower power, the Te flake not only has pristine crystal structure, which has lattice constant of 0.2 nm measured along {$1\bar{2}10$} direction, but also the structures with laser-induced defects, seeing the organ and light blue dash rectangles in Figure 4c, respectively. In the defect area, some Te atoms are removed by laser irradiation, resulting in the formation of vacancies in materials. The lattice constant increases to ~0.32 nm, which is consistent with the lattice of Te nanocrystals [28]. The variation of atom structures is related to the heating effect induced by laser [28,32,33]. In addition, DFT calculations have predicted that tellurene has large anisotropic lattice thermal conductivity [34], which may lead to anisotropic absorption of heat and formation of vacancies along a certain direction (shown in Figure 4c). Thus, lower power is able to generate some defects while remaining pristine crystal structure. In order to confirm the changes of element composition of Te flake after the laser irradiation, the EDS mapping measurement was performed using STEM and shown in Figure 4d,e. In the ablation area, high-power laser removes the Te materials and only left little oxides, which are caused by the oxidization of the supporting carbon film. However, outside the ablation area, the flake mainly contains Te and also O atoms, which are introduced by the laser irradiation. Especially, at the edge of the ablation hole, more Te and O atoms are demonstrated, revealing the formation of crystal and amorphous materials with oxides, as Figure 4d,e show. The line-scan EDS (Figure 4f) also proves the variations of element composition. Thus, combining the evolutions of atom structure, surface morphology, and Raman spectra, the laser is capable of inducing defects, oxides, and amorphous materials in the Te flake by adjusting the laser power and irradiation time, providing the gorgeous applications in controlled electrical properties of materials and corresponding nano-devices.

**Figure 5:**
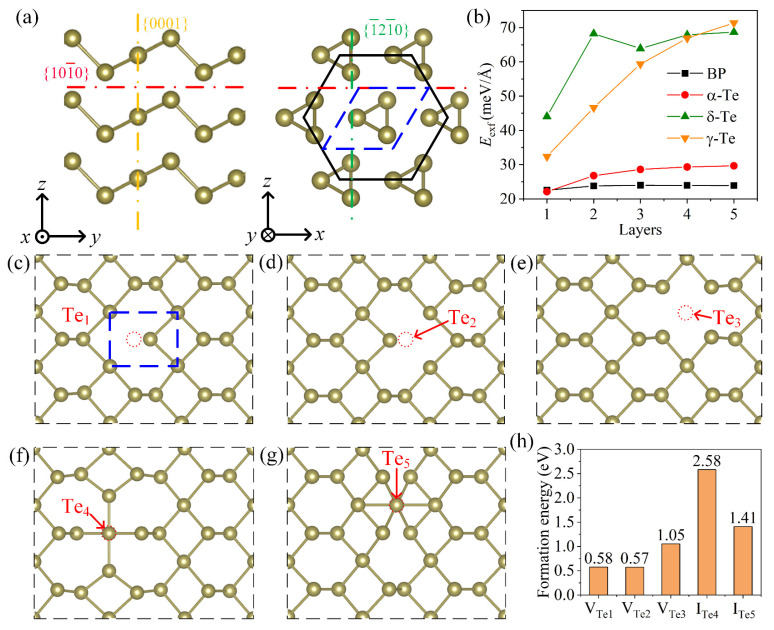
Exfoliation of Te flake and formation of defect. (**a**) atom structure of Te flake; (**b**) exfoliation energy of cutting along the {$10\bar{1}0$}, {0001}, and {$\bar{1}2\bar{1}0$} planes; relaxed atom structures with three vacancies V_Te1_ (**c**), V_Te2_ (**d**), and V_Te3_ (**e**) and two interstitials I_Te4_ (**f**) and I_Te5_ (**g**); (**h**) the formation energy of five possible defects.

DFT calculations were performed to explain the mechanism of laser-induced modification in Te flake (details are shown in Materials and Methods). Pervious experimental and simulated results showed Te flakes with thicknesses of 20–23 nm have layer number of ~100 [23,24,25,26]. Hence, the atom structure of the Te flake can be regarded as bulk tellurium, which is shown in Figure 5a. The bulk tellurium has a hexagonal cell with helical chains of Te atoms at the center and corners. Three phase of 2D Te, α-Te, δ-Te, and γ-Te, can be exfoliated from the bulk tellurium by cutting along the {$10\bar{1}0$}, {0001}, and {$\bar{1}2\bar{1}0$} planes, respectively [35]. Here, the exfoliation energy *E*_exf_ of tellurium was calculated to give a glimpse of the ablation of Te flake (Details are shown in Materials and Methods). Figure 5b exhibits the exfoliation energy of α-Te, δ-Te, and γ-Te with different layers and (The exfoliation energy of 2D BP was calculated as a reference). Furthermore, 2D BP has exfoliation energy of 24 meV/Å^2^, which is consistent with previous results [29] and proves the validity of the calculation method. The calculation results demonstrate the exfoliation of Te flake along the {$10\bar{1}0$} plane is much easier than other two directions, especially for the thicker exfoliated 2D layers. Hence, under the high laser power, all bonds are broken so that the Te atoms are removed from the flakes. However, laser with low power partially destroy the atom structures, resulting in the deformation of the materials and formation of nano-structures, which may cause the protrusions on the surface, as Figure 2d shows. As low power can also induce defects in the flakes, taking the monolayer α-Te as an example, formation energy of defects was calculated (see Materials and Methods). Since the primitive cell (the blue dash rectangles shown in Figure 5c) has three Te atoms, three types of vacancies (V_Te1_, V_Te2_, and V_Te3_) and two interstitials (I_Te4_ and I_Te5_) were simulated. Figure 5c–g show the relaxed atom structures with these five possible native defects. Figure 5h gives the corresponding formation energy. Comparing to the interstitial, formation of vacancy requires lower energy, especially for the vacancies of the Te_1_ and Te_2_ atoms. Hence, after laser irradiation, the predominate defects in the Te flakes are vacancies. In addition, since the formation energies of vacancies V_Te1_ and V_Te1_ are lower than V_Te3_, the heating induced by laser may cause the disappearance of part of the Te atoms and generation of vacancies along a certain direction, as Figure 4c shows. Such vacancies will promote the migration of metal ions or oxygen vacancies in materials, so that modifying the electrical properties of martials and nano-devices.

### 3.3. Application of Laser-Irradiated Te Nano-Flakes in Volatile Memristor

**Figure 6:**
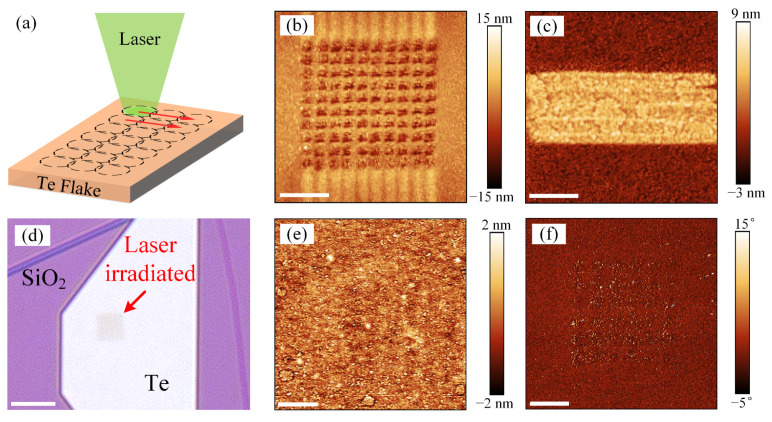
Laser patterning of a Te flake with thickness of 25 nm. (**a**) schematic of the laser pattering process, the dash cycles and red arrows represent the locations and moving directions of laser spots, respectively; laser-induced hole array (**b**) and increase of thickness (**c**) in the Te flake; optical (**d**), AFM height (**e**), and AFM phase (**f**) images of laser-irradiated Te flake without damage of surface, the scale bars in optical images and AFM images are 5 μm and 1 μm, respectively;.

Here, to investigate the influence of laser-induced modification of the electrical properties of materials and corresponding performance of nano-devices, laser patterning of Te flakes was processed using the mapping mode of Raman spectrometer by varying the irradiation time, power, and step of movement, as Figure 6a shows. A Te flake was patterned by the laser (the power was 0.57 mW, the irradiation time was 1 s, and the 100× objective lens was used) with steps of 300 nm, resulting in hole array on the surface (see Figure 6b). Moreover, due to the limited number of filters in Raman spectrometer, the objective lens to 50× was used to increase the size of laser spot for slightly decreasing the power density. The Te flake was exposed to laser for 30 s so that the thickness of the flake was increased due to the formation of nano-structure. Previous research has proved that defects and oxides in 2D materials can promote the migration of metal ions or oxygen vacancies in materials to form the conductive filament, which is the foundation of the electrical switching performance of memristor [36,37]. Here, to investigate the influence of laser-induced defects and oxides on the electrical properties, this paper concentrates on the performance of two terminal vertical memristor. Since the high-performance memristors require excellent contact between 2D martials and electrodes, suitable laser power, irradiation time and mapping parameters are chosen to avoid significant modification of surface morphology. For example, a Te flake with thickness of 25 nm was patterned by laser with power of 0.49 mW (using 100× objective lens) and time of 15 s, using a scanning step size of 500 nm in *x* and *y* directions. Parameters of laser power and irradiation time are both critical to performance the laser modification. The scanning step size of 500 nm in x and y directions was chosen according to the size of laser spot to ensure that the surface of material was completely irradiated. Figure 5d shows the optical image of a laser-irradiated Te flake. A clear change of color contrast in laser exposed area represents the surface modification, which results in the variation of optical properties of the Te flake. Figure 5e,f show the corresponding height and phase images of laser-irradiated surface using tapping mode of AFM. It can be seen that, after the laser patterning, the surface roughness has negligible changes. However, the changes of phase have proved the successful modification, which can be attributed to the laser-induced defects and oxides. Furthermore, to prove the effects of laser patterning with low power on atom structure, the Raman spectra were given in Figure S3. Similar to the results showed in Figure 3, after the laser irradiation, the intensities of the three characteristic peak increase without new peaks observed, indicating that laser-induced defects and oxides have influence on the atom structures. The variation of the Raman spectra confirms the laser-induced modification of Te flakes again. Hence, laser patterning with low power and long irradiation time can result in the defect and oxide in the Te flakes without damage of surface and gives opportunity for the development of memristor.

**Figure 7:**
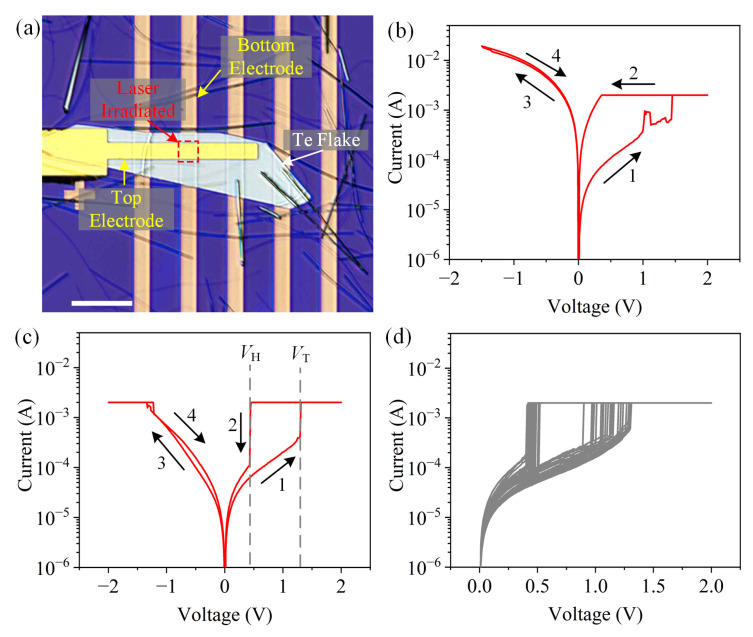
Volatile memristor based on laser-irradiated Te flake. (**a**) Optical image of two-terminal vertical nano-devices, scale bar is 20 μm; typical I–V curves of devices based on pristine (**b**) and laser-irradiated flakes (**c**). The black arrows label the voltage sweep order. (**d**) The endurance test of 30 cycles.

Two-terminal vertical nano-devices were fabricated via electron-beam lithography and e-beam evaporation (Details are shown in Materials and Methods). The Te flakes were irradiated by laser using the mapping mode of Raman spectrometer before the fabrication of top electrodes (the parameters of laser modification are same with the process shown in Figure 6d–f). Figure 7a shows the optical image of two vertical nano-devices based on the pristine and laser-irradiated Te (in the red dotted rectangle), respectively. To investigate the switching performance, the devices were swept by direct current (DC). The voltage is defined as the potential difference between the top electrode (Ti) and the bottom electrode (Au), and an electric field from Ti to Au was induced under positive voltage bias and vice versa. As Figure 7b presents, a positive voltage sweep (from 0 to 2 V) with compliance current I_CC_ of 2 mA sets the devices based on pristine Te from the HRS to LRS (seeing the arrow 1 and arrow 2, respectively). However, as the negative voltage was applied (from 0 to −1.5 V), the resistance of the device still maintains LRS and cannot be reset back to HRS (seeing the arrow 3 and arrow 4, respectively). This performance means that the device is broken by applied positive voltage [6]. Hence, the device based on pristine Te has no properties of memristor. In contrast, for the laser-irradiated Te (see Figure 7c), the abrupt switching from HRS to LRS (arrow 1) is observed when the voltage surpasses a threshold voltage *V*_T_ of 1.29 V (*I*_CC_ = 2 mA). As the voltage decreases lower than a hold voltage *V*_H_ of 0.43 V, the device is reset back to HRS (arrow 2), indicating that the device based on laser-irradiated Te is a digital-type volatile memristor. As the negative voltage applied, no further obvious decrease of the current (no Reset process) is exhibited. Hence, the switching performance is unipolar instead of bipolar [38]. Moreover, there is no symmetrical switching behavior during the negative voltage applying. It could be attributed to the electrochemical metallization mechanism (ECM) of memristor [39]. The positive voltage oxidizes the active Ti atoms from top electrode. Then, Ti cations migrate towards the bottom electrode and are reduced, leading to the formation of conductive filaments. However, as the bottom electrode has no active Ti atoms, the negative voltage cannot induce the migration of metal cations from the bottom electrode to top electrode and formation of filaments [40]. To confirm the volatile switching performance, a low voltage (0–0.1 V) was applied on the memristor before and after the positive voltage sweep. Figure S4 demonstrates the current state: the devices are both at the HRS with similar current before and after the positive voltage sweep, indicating the volatile behavior of memristor. To further reveal the stability of the volatile memristor based on laser-irradiated Te, an endurance test was carried out. As Figure 7d shows, the memristor demonstrates a relatively stable performance of volatile switching during the endurance test. In addition, different devices based on laser-irradiated Te all exhibit behavior of volatile memristor (Figure S5). However, the stability of memristor is based on laser-irradiated Te’s need to be improved in next investigation. Hence, according to the above investigation, it can be concluded that the laser-induced vacancy defects and oxides in Te flake promote the formation of the conductive filament which is the necessity of memristor. The memristor based on the laser-irradiated Te flake provides enormous potential applications in neuromorphic computing, hardware security, and access device [39].

## 4. Conclusions

Effects of laser irradiation on Te are revealed by varying laser power and irradiation time in air atmosphere. High power laser can totally ablate, remove, and further thin the flakes. The laser ablation and thinning provides ways to develop laser-patterned transistor and photodetectors. Under the low laser power, despite no ablation and thinning being observed in Te, amorphous materials, oxides, and defects are induced. Besides, by prolonging irradiation time, the low power laser results in nano-protrusions on the surface of the Te flake. Such protrusions can be attributed to the deformation of materials and the formation of nano-structures. This phenomenon can be explained by the calculated exfoliation energy, which has proved that the Te flake is more easily broken along the direction of Te chains than the other direction. Moreover, the simulations also demonstrate that the predominate defects in the Te flakes are vacancies, which are benefit for the movement of metal ions or oxygen vacancies in materials to form conductive filament for memristor. With the foundation of the laser-induced defects and oxides, device based on laser-irradiated Te flake exhibits the performance of digital-type volatile memristor, which is not exhibited in pristine flakes. The resistive switching in memristor indicates the formation of conductive filaments, since the defects and oxides promote the migration of metal ions. Hence, laser-irradiated Te opens a route to the development of memristor and next-generation computing system.

## Data Availability

The data presented in this study are available on request from the corresponding author.

## Supplementary Materials

The following are available online at https://www.mdpi.com/article/10.3390/ma16020738/s1, Figure S1 The variations of fitting positions and intensities of Raman peaks for Te flakes with different -thicknesses; Figure S2 AFM images (top inset images) of Te flakes after low-power laser (0.72 mW) irradiation using a 50× objective lens and the corresponding height profiles (bottom inset images, the positions are shown by the blue dotted lines in the top inset images); Figure S3. Changes of Raman spectra in Te a flake after laser patterning. Figure S4. The current states of a memristor before and after the positive voltage sweep. Figure S5. Typical I–V curves of 5 volatile memristors based on laser-irradiated Te flakes.
