# Supplementary material for: Direct Laser Irradiation and Modification of 2D Te for Development of Volatile Memristor
Source: Materials (Basel). 2023 Jan 12;16(2):738. doi: 10.3390/ma16020738 (PMC9862747; doi:10.3390/ma16020738)
Supplement: Supplementary file 1 [file materials-16-00738-s001.zip › materials-2082120-supplementary.pdf]

Supplementary Materials

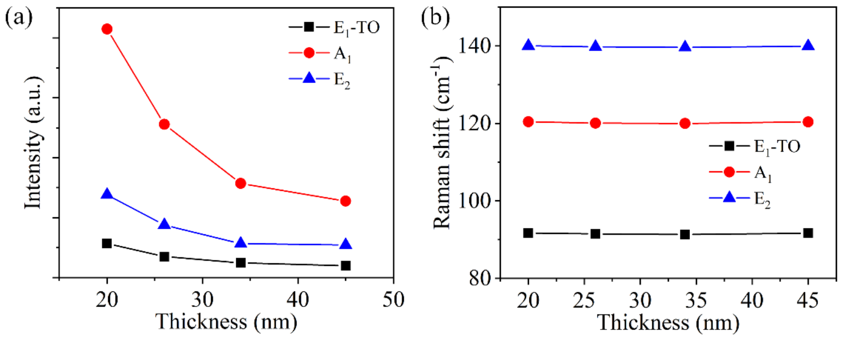

Figure S1 The variations of fitting positions and intensities of Raman peaks for Te flakes with different thicknesses. (a) fitting intensities; (b) fitting positions.

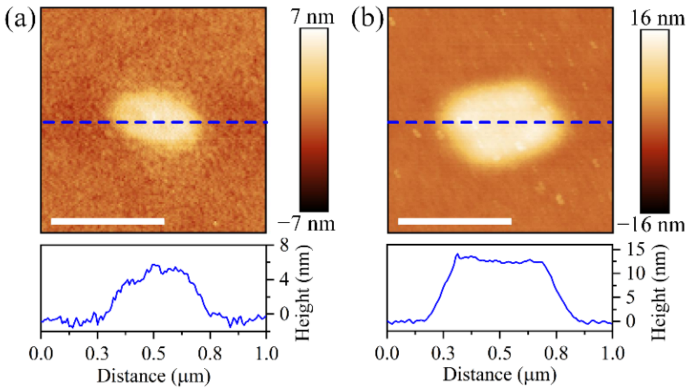

Figure S2 AFM images (top inset images) of Te flakes after low-power laser (0.72 mW) irradiation using a 50× objective lens and the corresponding height profiles (bottom inset images, the positions are shown by the blue dotted lines in the top inset images), scale bar is 500 nm. (**a**) irradiation time is 60s; (**b**) irradiation time is 120s;

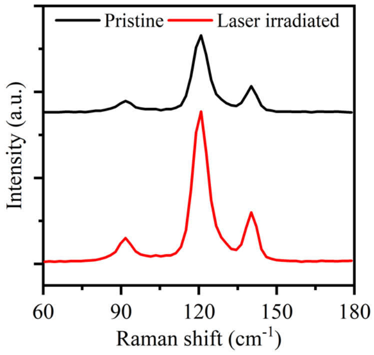

Figure S3. Changes of Raman spectra in a Te flake after laser patterning

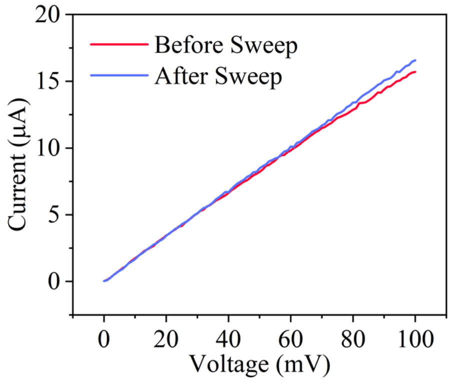

Figure S4. The current states of a memristor before and after the positive voltage sweep

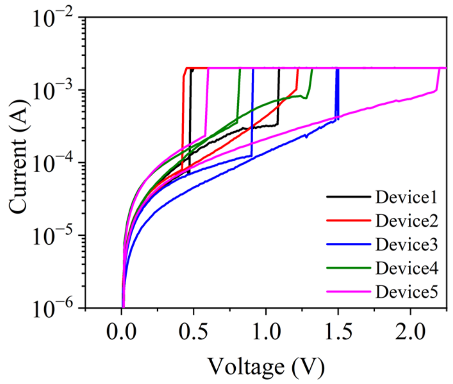

Figure S5. Typical I–V curves of 5 volatile memristors based on laser-irradiated Te flakes
